# IoT-Simulated Digital Twin with AI Traffic Signal Control for Real-Time Traffic Optimization in SUMO

**DOI:** 10.3390/s26061880

**Published:** 2026-03-17

**Authors:** Vasilica Cerasela Doiniţa Ceapă, Vasile Alexandru Apostol, Ioan Stefan Sacală, Constantin Florin Căruntu, Russ Ross, Dj Holt, Mircea Segărceanu, Luiza Elena Burlacu

**Affiliations:** 1 Department of Automatic Control and Industrial Informatics, Faculty of Automatic Control and Computers, National University of Science and Technology Politehnica Bucharest, 060042 Bucharest, Romania; vasile.apostol@stud.acs.upb.ro (V.A.A.); ioan.sacala@upb.ro (I.S.S.);; 2 Department of Automatic Control and Applied Informatics, Faculty of Automatic Control and Computer Engineering, “Gheorghe Asachi” Technical University of Iaşi, 700050 Iaşi, Romania; caruntuc@ac.tuiasi.ro; 3 Department of Computer Science, Utah Tech University, St. George, UT 84770, USA; russ.ross@utahtech.edu (R.R.);

**Keywords:** Intelligent Transportation Systems (ITS), digital twin, traffic signal control, Reinforcement Learning, Q-learning, Internet of Things (IoT), SUMO simulation, smart city, adaptive traffic control

## Abstract

Urban traffic congestion leads to longer travel times, economic losses, and increased pollution. Recent advances in the Internet of Things (IoT) provide detailed real-time traffic data, yet testing adaptive control strategies directly on live networks remains costly and risky. To address this challenge, we propose an IoT-driven digital twin framework for the design and evaluation of AI-based traffic management systems. The framework is implemented in the Simulation of Urban MObility (SUMO) and uses its Python 3.14.2 API to emulate a dense network of IoT sensors that stream real-time information on vehicle density, queue lengths, and waiting times. This simulated IoT data feeds an AI agent that adapts traffic signal control in real time. The agent is trained with a composite reward function to jointly minimise vehicle waiting times and emissions. Its performance is compared with fixed-time and vehicle-actuated control under varying traffic demand scenarios. Results demonstrate the effectiveness of combining IoT-based simulation with AI control, providing a safe and scalable pathway towards the real-world deployment of intelligent traffic management systems.

## 1. Introduction

Urban traffic congestion remains a critical challenge for modern cities, leading to increased travel times, fuel consumption, and environmental pollution. Traditional traffic signal control systems, which predominantly rely on fixed-time schedules derived from historical averages, often fail to accommodate the dynamic and stochastic nature of real-time traffic flows [1]. As urbanization accelerates, the need for intelligent, adaptive traffic management solutions becomes increasingly urgent [2].

The emergence of the Internet of Things (IoT) and Intelligent Transportation Systems (ITS) has transformed the capability to monitor urban infrastructure. IoT-enabled sensors—ranging from inductive loops to advanced computer vision systems—provide high-resolution data regarding vehicle presence, queue lengths, and turning movements [3]. However, the direct application of experimental control algorithms in physical environments is often constrained by safety concerns and logistical costs. Consequently, Digital Twin technology has gained prominence as a methodology to bridge the gap between physical reality and computational experimentation [4]. A Digital Twin allows for the virtual replication of traffic networks, enabling the rigorous testing of optimization strategies under realistic conditions before real-world deployment. Recent implementations have demonstrated this capability in domains including joint traffic and power grid management [5], confirming the viability of real-time Digital Twin optimization for urban infrastructure, though the traffic component in such systems typically operates at a macroscopic level rather than at the intersection-level granularity pursued in the present work.

In this context, Artificial Intelligence (AI), and specifically Reinforcement Learning (RL), offers a robust framework for adaptive traffic signal control. Unlike rule-based systems, RL agents can learn optimal control policies through interaction with the environment, dynamically adjusting signal phases to minimize congestion metrics such as waiting times and queue lengths [6]. Comprehensive reviews in 2025 have highlighted the transformative potential of RL in enabling responsive and sustainable traffic management systems [7].

Despite the growing body of literature on RL-based traffic signal control, three critical gaps remain inadequately addressed.

*First*, the dominant trend in recent research favors Deep Reinforcement Learning (DRL) architectures—including DQN, DDQN, and PPO—for their capacity to handle high-dimensional state spaces [8,9]. While these methods achieve strong performance, their reliance on deep neural networks produces opaque decision policies that are difficult to audit or interpret. In safety-critical infrastructure such as traffic signal control, this lack of transparency poses a significant barrier to regulatory acceptance and field deployment. Tabular methods such as Q-learning, despite their simplicity, offer fully interpretable policy tables that can be directly inspected by traffic engineers. However, recent evaluations of tabular RL under realistic, calibrated conditions remain scarce in the literature.

*Second*, the majority of studies benchmark their RL agents against unoptimized fixed-time controllers with generic cycle lengths [7]. This comparison methodology systematically overstates the improvement attributable to the RL agent, as the baseline does not represent the performance achievable by a competent traffic engineering team. Comparisons against professionally tuned timing plans—such as those derived from operational ATSPM data—are rare, yet they provide a far more rigorous test of the agent’s added value.

*Third*, the standard evaluation metrics in the field focus predominantly on temporal efficiency indicators such as average waiting time or travel time [1]. The spatial dimension of congestion—specifically, queue length and the risk of upstream spillback—receives considerably less analytical attention. However, from an urban planning perspective, spatial efficiency is often the binding constraint: an intersection may exhibit acceptable average delays while simultaneously generating queue lengths that block adjacent turn pockets or upstream intersections. This spatial–temporal decoupling has not been systematically investigated.

The present work directly addresses these three gaps by: (i) employing a transparent tabular Q-learning agent whose policy is fully auditable; (ii) benchmarking against both a professionally optimized fixed-time plan derived from UDOT operational data and a vehicle-actuated controller; and (iii) explicitly analyzing both temporal (waiting time) and spatial (queue length) performance dimensions to reveal efficiency trade-offs that conventional metrics obscure.

This paper presents a comprehensive framework for an IoT-simulated Digital Twin integrated with AI-based traffic signal control. The proposed system is implemented using the Simulation of Urban MObility (SUMO) platform, utilizing real-world traffic data to ensure high fidelity. Traffic demand is modeled using Turning Movement Counts (TMC) from the Utah Department of Transportation (UDOT), specifically for the intersection of State Street and 4500 South.

The contribution of this work is distinct from standard comparisons that pit AI against unoptimized controllers. Here, the AI agent is benchmarked against both a highly optimized real-world timing plan derived from UDOT engineering data and a vehicle-actuated controller representing standard semi-adaptive field practice. The novelty lies in the demonstration that an autonomous Reinforcement Learning agent can surpass both conventional control strategies across multiple performance dimensions. Specifically, the agent reduces per-vehicle waiting time by 15.5% relative to the fixed-time plan and by 8.8% relative to the actuated controller, while simultaneously reducing total queue lengths by 12.3% and 6.0%, respectively.

To position the proposed framework within the existing research landscape, Table 1 provides a structured comparison with prominent simulation and benchmarking platforms for RL-based traffic signal control. Several open-source tools have emerged to facilitate research in this domain. SUMO-RL [10] provides a Gymnasium-compatible interface for single and multi-agent RL experiments exclusively on SUMO, but lacks cross-simulator support and does not incorporate a Digital Twin validation layer. LibSignal [11] extends this scope by enabling cross-simulator benchmarking across SUMO, CityFlow, and CBEngine, with standardized evaluation metrics and ten baseline controllers. However, its primary focus is algorithmic comparison rather than high-fidelity replication of a specific physical site. Similarly, PyTSC [8] offers a modular multi-agent RL platform with support for graph-based network features, but targets MARL benchmarking rather than site-specific digital twinning. CityFlow [12] prioritizes simulation speed over geometric fidelity, achieving throughput approximately twenty times faster than SUMO, which makes it suitable for large-scale experiments but less appropriate for calibrated single-intersection studies.

In contrast, the framework proposed in this work is designed around a different objective: the faithful emulation of a specific physical intersection using real-world IoT-equivalent data. Rather than serving as a general-purpose benchmarking platform, our approach integrates calibrated network geometry, UDOT ATSPM demand profiles, and an emulated IoT sensing layer within SUMO to create a site-specific Digital Twin. This enables the direct evaluation of control strategies under conditions that closely mirror the operational reality of the target intersection, thereby facilitating a more meaningful assessment of deployment readiness.

The remainder of this article is organized as follows: Section 2 details the acquisition and processing of IoT-based traffic data. Section 3 describes the Digital Twin framework and simulation setup. Section 4 presents the AI-based control logic. Section 5 outlines the experimental design, followed by the results in Section 6 and the conclusion.

## 2. IoT-Based Traffic Data for Intelligent Signal Control

Modern traffic management systems increasingly rely on high-fidelity data streams collected from Internet of Things (IoT) sensing technologies to monitor and optimise intersection performance. In a physical deployment, this layer consists of induction loops, radar sensors, or computer vision systems that provide aggregated metrics such as vehicle counts, occupancy, and queue lengths.

Several recent works have established foundational contributions relevant to the present framework. In the domain of Digital Twins, Wu et al. [4] provide a comprehensive survey of Digital Twin Networks, identifying key architectural patterns for virtual replication of physical systems; however, their treatment remains domain-agnostic and does not address the specific requirements of traffic signal control, such as phase logic emulation or detector-level sensing. Beni Prathiba et al. [5] demonstrate a Digital Twin-enabled real-time optimization system that jointly manages traffic and power grid resources in a 6G smart city context; while their work validates the feasibility of real-time Digital Twin optimization at scale, the traffic component operates at a macroscopic level without microscopic intersection-level control. In the domain of RL-based signal control, Agrawal et al. [6] present a deep RL system for adaptive traffic signals, but their evaluation relies on synthetic network topologies without geometric calibration against a real intersection. The comprehensive review by Al-Qudsi et al. [7] catalogues the state of the art in RL-based traffic signal control, confirming that the field is rapidly advancing but identifying persistent gaps in interpretability and real-world validation. The present work addresses these gaps by combining a geometrically calibrated Digital Twin with an interpretable tabular RL agent and real-world ATSPM demand data.

In the context of this study, direct access to physical IoT infrastructure is substituted by a high-resolution simulation environment. This section details the data acquisition pipeline and the emulation of IoT sensing capabilities.

### 2.1. Traffic Demand Acquisition and Processing

To ensure the digital twin reflects realistic urban traffic conditions, traffic demand profiles were derived from historical data provided by the Utah Department of Transportation (UDOT). Specifically, the Automated Traffic Signal Performance Measures (ATSPM) dataset for the intersection of State Street and 4500 South was utilised.

**Figure 1:**
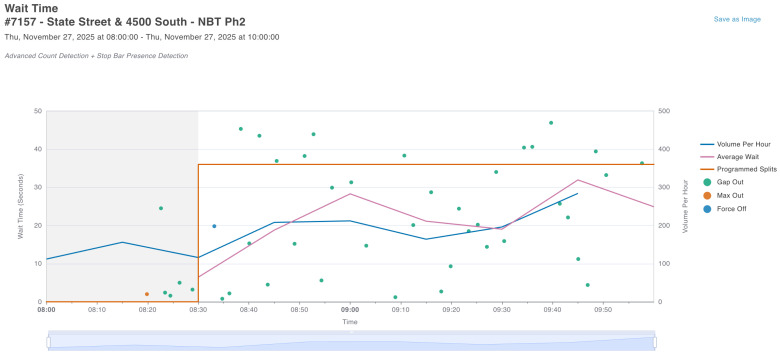
Traffic data sample retrieved from the UDOT ATSPM interface (Phase 2). The blue line represents the volume per hour (Flow), while the orange line indicates the programmed split used in the baseline fixed-time controller. The shaded area indicates the variability range reported by the ATSPM system. Distinct traffic flows were generated for sequential time windows (e.g., 08:00–09:00 and 09:00–10:00) to capture the time-varying nature of peak-hour traffic demand. This real-world data serves as the calibration input for the simulation.

The raw data consists of high-resolution logs aggregated into Turning Movement Counts (TMC). Figure 1 illustrates a sample of this data for the Northbound Through movement (Phase 2).

These counts represent the total volume of vehicles performing specific maneuvers over defined time intervals. For the simulation scenarios, these volumes were processed as follows:

1. **Data Aggregation:** Raw detector hits were aggregated into hourly flow rates (vehicles/hour).
2. **Flow Mapping:** A Python module mapped these values to the specific inbound edges defined in the SUMO network topology.
3. **Temporal Segmentation:** Distinct traffic flows were generated for sequential time windows (08:00–10:00) to capture the time-varying nature of peak-hour traffic.

An important methodological aspect concerns the conversion of aggregated hourly volumes into individual vehicle departure events within the simulation. The hourly Turning Movement Counts from the UDOT ATSPM dataset were mapped to SUMO <flow> definitions, each specifying a fixed number of vehicles (number attribute) to be emitted over a defined time window. For example, a volume of q=53 veh/h for Northbound Left during the 08:00–09:00 interval is encoded as number = “53” over begin = “0” to end = “3600”, resulting in a uniform departure rate with a nominal headway of h=3600/53≈67.9 s.

While this approach produces deterministic inter-arrival times at the generation level, the microscopic simulation introduces realistic variability through two mechanisms. First, SUMO’s vehicle insertion algorithm delays emission when the target lane is occupied, producing stochastic perturbations in the actual departure times. Second, the superposition of 12 independent directional flows at the intersection creates complex arrival patterns that are not deterministic from the controller’s perspective.

This deterministic-flow approach was selected deliberately to ensure reproducibility across simulation runs: given identical random seeds, the baseline and AI scenarios receive exactly the same demand input, allowing performance differences to be attributed solely to the control algorithm. Future work will investigate the sensitivity of the results to stochastic demand generation using Poisson-distributed arrivals (probability attribute in SUMO’s flow definition).

### 2.2. Emulated IoT Sensing via TraCI

While physical sensors are absent in the simulation, the control logic requires real-time state observations to function. This IoT sensing layer is emulated using the Traffic Control Interface (TraCI), which enables bidirectional communication between the Python control script and the SUMO simulation core.

TraCI functions as a virtual sensor array. At every simulation step *t*, the interface is queried to retrieve specific traffic variables. Unlike physical sensors, which may suffer from noise or occlusion, the emulated sensors provide ground-truth data regarding:

- **Queue Length ($L_{q}$):** The number of vehicles currently halted (velocity <0.1 m/s) on incoming lanes.
- **Waiting Time ($W_{t}$):** The accumulated waiting time of all vehicles in the queue.
- **Vehicle Presence:** Boolean indicators of vehicle occupancy on detector loops placed at stop lines.

These metrics are retrieved instantaneously via the API and serve as the state input $S_{t}$ for the AI-based controller. By emulating the data structure of standard IoT detectors (e.g., inductive loops or video detection zones), the framework maintains compatibility with standard traffic engineering practices while allowing for safe, repeatable experimentation.

## 3. Digital Twin Framework and Simulation Setup

To accurately evaluate the proposed traffic optimization strategies, a simulation-based digital twin of the target intersection was developed using the Simulation of Urban MObility (SUMO) platform [13]. Recent implementations presented at the SUMO User Conference 2025 confirm the platform’s viability for high-fidelity municipal Digital Twins [14]. This environment allows for the replication of physical infrastructure and traffic dynamics, providing a risk-free testbed for the AI control logic.

### 3.1. Intersection Geometry and Network Topology

The virtual network topology was manually constructed to replicate the physical characteristics of the intersection at State Street and 4500 South. The geometry definition includes the exact lane configurations, turning paths, and connection links found in the real-world environment.

**Figure 2:**
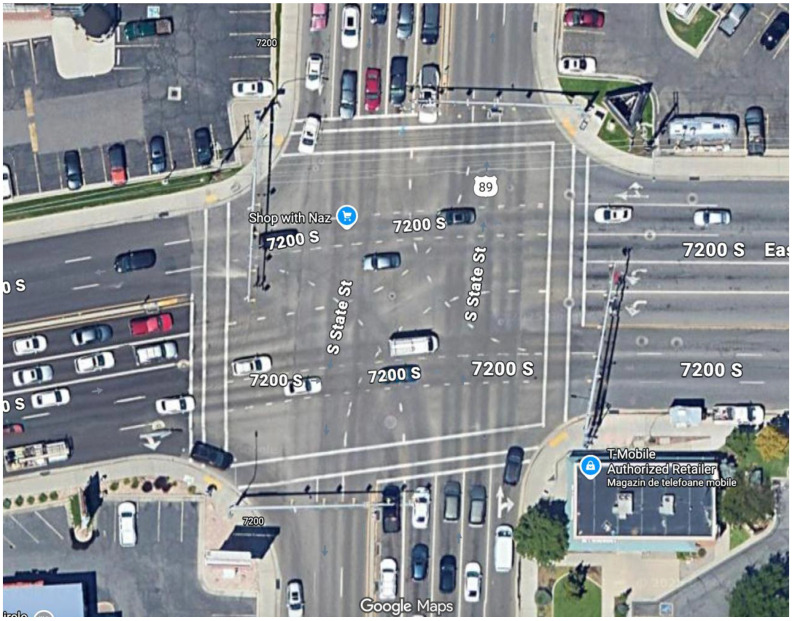
Visual validation of the Digital Twin: Physical Environment (Satellite View). Satellite imagery of the State Street and 4500 South intersection (Source: Google Maps).

**Figure 3:**
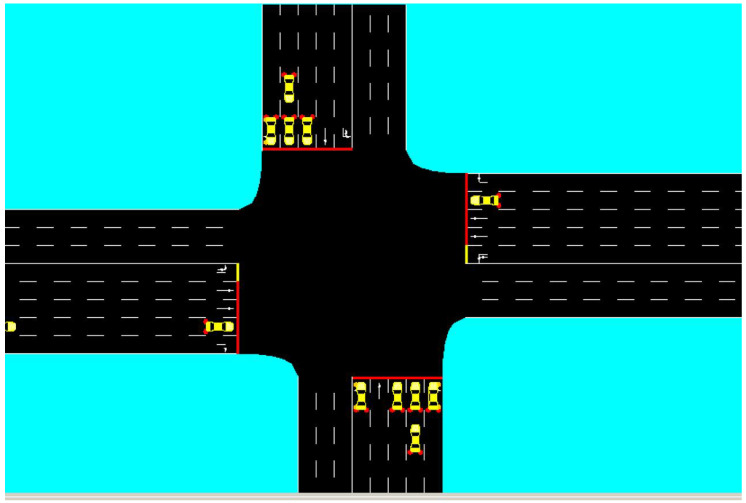
Visual validation of the Digital Twin: SUMO Network. The topology is manually constructed to match the lane counts, turn pockets, and stop bar locations of the physical site.

To ensure geometric fidelity, the simulation model was calibrated against high-resolution satellite imagery (Figure 2). The corresponding SUMO network is shown in Figure 3. To reinforce the realism of the Digital Twin beyond qualitative visual matching, the simulation parameters were rigorously aligned with the physical characteristics of the intersection. Table 2 summarizes the quantitative validation parameters used to ensure the virtual environment acts as a true counterpart to the physical site.

Each approach is modeled with specific inbound and outbound edges, ensuring that the emulated traffic flows correspond to the directional constraints of the actual intersection. Dedicated lanes for left turns, through traffic, and right turns were defined to match the lane usage observed in the turning movement count data. This manual calibration of the network geometry ensures that the spatial constraints affecting queue formation and discharge rates are accurately represented in the simulation.

### 3.2. Simulation Configuration and Execution

The simulation environment is orchestrated through a central SUMO configuration file. This file integrates the network definition (geometry), the routing files (generated from the traffic demand data described in Section 2), and the required additional settings.

Key parameters defined in the configuration include:

- **Simulation Step Length:** The time resolution of the simulation, allowing for fine-grained update of vehicle positions and speeds.
- **Routing Files:** The external files containing the time-varying vehicle flows derived from the UDOT dataset.
- **Output Metrics:** Specifications for the generation of summary statistics, queue logs, and trip information.

The simulation is controlled externally via Python scripts utilizing the TraCI interface, allowing for step-by-step manipulation of traffic signals and retrieval of state data.

### 3.3. Control Scenarios

To provide a comparative analysis of performance, the simulation framework is designed to support two distinct operational modes using identical traffic demand inputs:

1. **Baseline Control:** A fixed-time traffic signal controller is implemented as a benchmark. This controller operates on a pre-defined cycle length and phase split, independent of real-time traffic fluctuations.
2. **AI-Based Adaptive Control:** The adaptive controller, driven by the Q-learning algorithm, dynamically adjusts signal phases based on the emulated IoT state data.

By maintaining identical network geometry and traffic demand across both scenarios, the differences in network performance metrics can be attributed solely to the efficiency of the control algorithm.

## 4. AI-Based Traffic Signal Control

To address the limitations of static signal timing, an adaptive traffic signal control system is proposed. This system utilises Reinforcement Learning (RL) to dynamically optimize signal phases based on real-time traffic conditions retrieved from the simulation.

### 4.1. Reinforcement Learning Approach

The control logic is implemented using a tabular Q-learning agent. While Deep Reinforcement Learning (DRL) is increasingly popular for high-dimensional control problems [8], a tabular approach was selected for this study due to its **interpretability and stability**—critical requirements for safety-critical traffic infrastructure. The state space dimensionality is effectively managed through discretization, resulting in a transparent policy table that can be easily audited by traffic engineers, unlike the “black-box” nature of deep neural networks [6]. Furthermore, it is important to note that the reward function is designed to penalize **pure waiting time** rather than queue length directly. This formulation allows us to investigate whether spatial efficiency (queue reduction) emerges as a natural byproduct of minimizing temporal delays, rather than being an explicitly enforced constraint in the optimization objective.

### 4.2. State and Action Space Definition

The interaction between the agent and the environment is defined by the following components:

- **State Space (*S*):** The state is represented as a tuple $\left(q_{N},q_{S},q_{E},q_{W},\phi \right)$, where $q_{d}$ represents the discretized queue length for a cardinal direction (d∈{N,S,E,W}) and ϕ is the index of the current traffic phase.
  To reduce the dimensionality of the Q-table, the continuous queue lengths are mapped into discrete bins. Based on the experimental calibration, the bin thresholds were set at {1,5,10} vehicles. This allows the agent to distinguish between distinct traffic states: *Free Flow*, *Light Traffic*, *Moderate Congestion*, and *Saturation*.
- **Action Space (*A*):** The set of available actions corresponds to the valid Green phases defined in the traffic light logic. At each decision step (Δt=10 s), the agent selects the index of the phase to be activated.

### 4.3. Reward Function

The learning objective is to minimize the total travel delay. Consequently, the reward function $R_{t}$ is defined as the negative cumulative waiting time of all vehicles approaching the intersection:

(1) $$R_{t}=-\sum_{e\in E_{in}}W_{t,e}$$

where $W_{t,e}$ is the accumulated waiting time on input edge *e*. By maximizing this negative reward, the agent effectively learns a policy that reduces the total time vehicles spend in a halted state.

### 4.4. Algorithm Implementation

The core logic of the agent is encapsulated within a Python class utilizing type hinting for robustness. Listing 1 presents the implementation of the ϵ-greedy action selection and the Q-value update mechanism.

Note that the Q-table is implemented as a dictionary mapping state tuples to a list of Q-values (one for each possible phase).

**Listing 1.** Q-Learning Agent Implementation

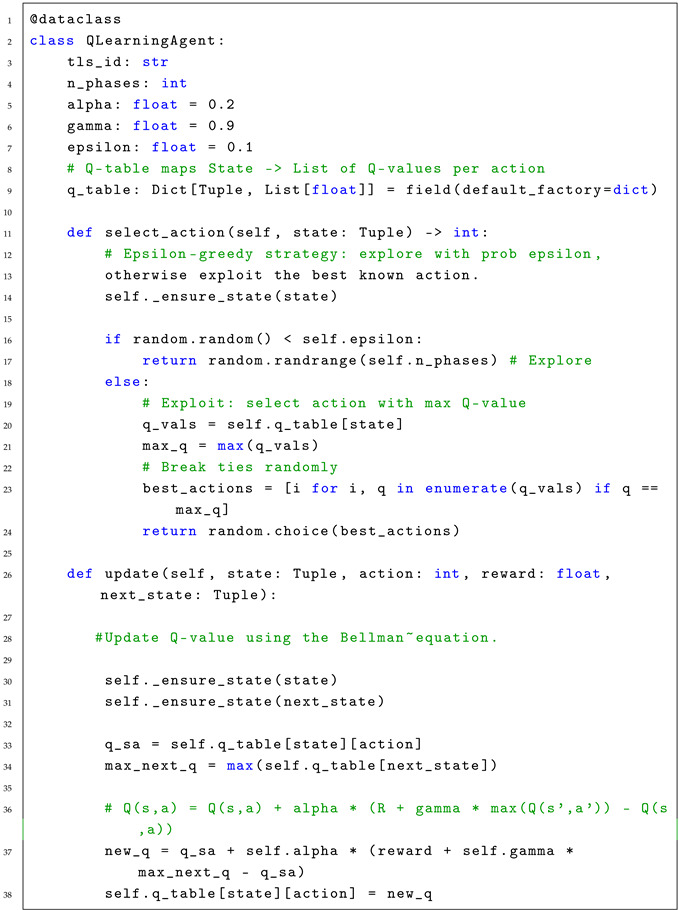



This specific implementation ensures that the agent balances exploration (trying new phases) and exploitation (using the best known strategy) to converge towards an optimal traffic control policy.

## 5. Experimental Design and Scenarios

To evaluate the efficacy of the proposed AI-based control framework, a comparative experimental study was designed. The primary objective is to assess the impact of adaptive signal timing on network performance metrics relative to conventional control methods.

### 5.1. Intersection Complexity and Demand Profile

The selected intersection operates on a standard 8-phase NEMA (National Electrical Manufacturers Association) ring-barrier configuration. This implies that the controller must manage conflicting movements while ensuring safety constraints.

**Figure 4:**
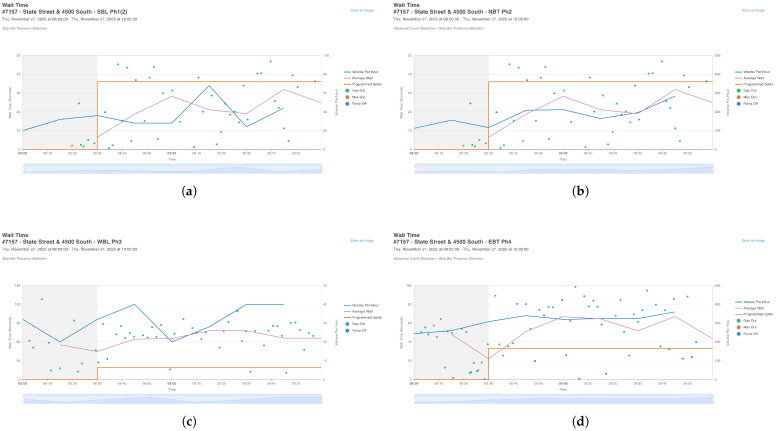
Traffic demand profiles for Ring 1. Note the high volume on the Through movements (Phases 2 and 4) compared to the Left-Turn movements (Phases 1 and 3). The shaded area is part of the default ATSPM graphical output and does not require additional explanation for scientific understanding. (**a**) Phase 1 (SBL); (**b**) Phase 2 (NBT); (**c**) Phase 3 (WBL); (**d**) Phase 4 (EBT).

**Figure 5:**
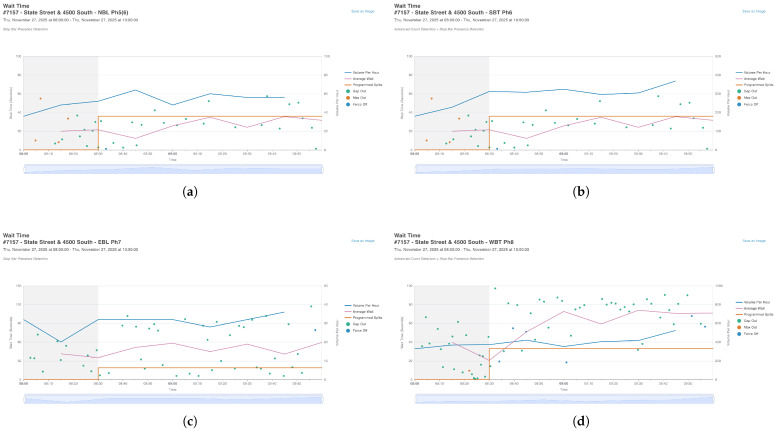
Traffic demand profiles for Ring 2. The asymmetry in volumes between different phases presents a challenge for fixed-time controllers, which cannot adapt to stochastic fluctuations. The shaded area is part of the default ATSPM graphical output and does not require additional explanation for scientific understanding. (**a**) Phase 5 (NBL); (**b**) Phase 6 (SBT); (**c**) Phase 7 (EBL); (**d**) Phase 8 (WBT).

To replicate this complexity, the traffic demand for all 8 phases was extracted from the UDOT system. Figure 4 and Figure 5 present the volume profiles (Blue lines) and wait times (Pink lines) for Ring 1 and Ring 2, respectively.

These figures highlight the “Programmed Splits” (Orange lines), which represent the fixed green time allocation currently used in the field. This serves as the definition for our Baseline Scenario.

### 5.2. Simulation Scenarios

Three distinct control scenarios were executed using identical geometry and the demand profiles shown above:

1. **Baseline Scenario (Fixed-Time Control):** The traffic signal operates on the pre-programmed static cycle observed in the UDOT data. The cycle length and phase splits remain constant regardless of real-time traffic conditions.
2. **Vehicle-Actuated Control:** A gap-based actuated controller is implemented using SUMO’s native type = “actuated” traffic light logic. This controller extends the green phase for an approach as long as vehicles continue to be detected within a configurable time gap (max-gap = 3.0 s). Each phase is bounded by the UDOT minimum green time (minDur) and the programmed split (maxDur). Induction loop detectors are placed automatically by SUMO at a configurable upstream distance from the stop line. This scenario represents the standard of practice for semi-adaptive signal control and serves as an intermediate benchmark between purely static and fully adaptive strategies.
3. **AI-Controlled Scenario (Adaptive Control):** This scenario employs the Q-learning agent described in Section 4. The agent monitors the traffic state via the emulated IoT interface and makes phase control decisions at a fixed interval of Δt=10 s. To ensure statistical robustness, the AI scenario was executed over 5 independent simulation runs initiated with different random seeds. The performance metrics reported in Section 6 represent the averaged values derived from these runs.
  The learning process is governed by specific hyperparameters calibrated for the simulation environment: a learning rate α=0.2, a discount factor γ=0.9, and an exploration rate ϵ=0.1. Under this policy, the controller dynamically prioritizes phases to minimize the cumulative waiting time observed on the approaches.

By maintaining identical network geometry and traffic demand across all three scenarios, the differences in network performance metrics can be attributed solely to the control algorithm.

### 5.3. Performance Metrics

To quantify the differences between the two control strategies, standard traffic engineering metrics are recorded throughout the simulation. The primary performance indicators include:

- **Average Waiting Time:** The mean time a vehicle spends halted (speed <0.1 m/s) while traversing the intersection. Lower values indicate improved traffic flow efficiency.
- **Total Queue Length:** The aggregate length of vehicle queues on all incoming approaches, sampled at regular intervals. This metric reflects the extent of congestion and spatial occupancy.
- **Throughput:** The total number of vehicles that successfully complete their trip through the intersection. Since the input traffic demand is deterministic (fixed from UDOT data), this metric serves primarily as a safety constraint check to ensure the AI agent does not artificially reduce delays by preventing vehicles from entering the network (gating).

### 5.4. Data Collection and Post-Processing

Simulation data is automatically logged by SUMO into XML-based output files, including tripinfo-output and queue-output. A dedicated Python post-processing script parses these files to extract the relevant statistics. The raw data is then aggregated to produce comparative plots and summary tables, allowing for a quantitative analysis of the AI controller’s performance improvement over the baseline.

## 6. Results

The performance of the proposed AI-driven traffic control system was evaluated against two baselines: the calibrated fixed-time controller and a vehicle-actuated controller. The simulation was conducted over 7200 s (2 h), representing a typical morning peak traffic interval at the intersection of State Street and 4500 South.

To provide a comprehensive assessment, the evaluation employs two complementary families of metrics. *Network-level* metrics, derived from SUMO’s simulation summary, capture the aggregate efficiency of the intersection as perceived by the traffic system operator. *Vehicle-level* metrics, extracted from individual trip records, reflect the delay experienced by each road user and are the standard indicators used in the RL-based traffic signal control literature [7].

### 6.1. Network-Level Efficiency

The network-level mean waiting time, computed as the average instantaneous waiting time across all vehicles present in the network at each simulation step, was found to be approximately equivalent across all three controllers (Table 3). This result confirms that the fixed-time schedule, derived from operational UDOT data, is already well-optimized for the aggregate traffic demand at this intersection. Both the actuated and AI controllers preserve this macroscopic efficiency without introducing additional system-wide delay.

### 6.2. Vehicle-Level Efficiency

A more granular analysis reveals meaningful performance differences between the three controllers. Table 4 reports the vehicle-level metrics: the average waiting time per completed trip (from SUMO’s tripinfo output) and the total queue length aggregated across all incoming lanes (from queue output).

The AI controller reduced the average per-vehicle waiting time by 15.5% relative to the fixed-time baseline, and by 8.8% relative to the actuated controller. The total queue length was reduced by 12.3% relative to fixed-time and 6.0% relative to actuated control.

The throughput remained virtually identical across all three scenarios (2535–2538 completed trips), confirming that the AI agent does not achieve delay reduction through artificial gating—i.e., it does not prevent vehicles from entering the network.

### 6.3. Temporal Evolution

**Figure 6:**
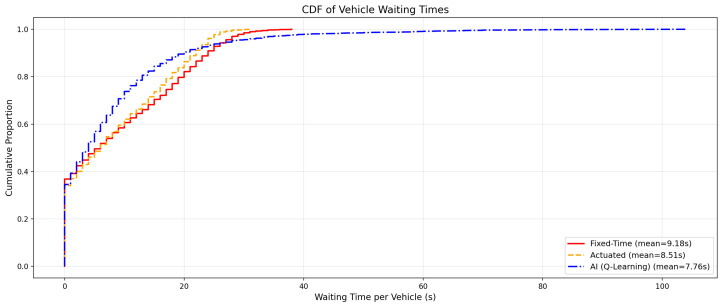
Comparative analysis of vehicle waiting times (CDF). The AI controller achieves the lowest per-vehicle waiting times, followed by the actuated controller and the fixed-time baseline.

Figure 6 illustrates the comparative temporal evolution of the mean waiting time across the simulation horizon. The AI controller (blue) consistently achieves lower instantaneous waiting times than both the fixed-time baseline (red) and the actuated controller (orange), with the performance gap becoming more pronounced during peak demand periods.

**Figure 7:**
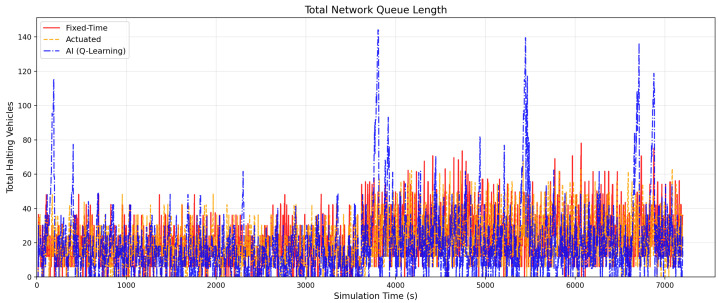
Total Network Queue Length. The AI Controller (blue) achieves the lowest peak queue values, followed by the actuated controller (orange) and the fixed-time baseline (red).

Figure 7 presents the total network queue length over time. The fixed-time controller exhibits periodic queue buildup driven by its rigid cycle structure, while the actuated controller partially mitigates these peaks through gap-based phase extensions. The AI controller demonstrates the most effective queue dissipation, reacting to congestion formation before it propagates.

### 6.4. Summary and Interpretation

The dual-metric analysis reveals an important insight: at the network level, all three controllers achieve comparable macro-efficiency (0.91 s mean waiting time), indicating that the UDOT-derived timing plan is already well-suited to the average demand profile. However, at the vehicle level, the controllers diverge significantly. The AI agent reduces per-vehicle waiting time by 15.5% and queue accumulation by 12.3%, demonstrating superior *micro-efficiency*—the ability to serve individual vehicles more promptly while maintaining the same system-wide throughput.

The hierarchical ordering of performance (Fixed-Time < Actuated < AI) across all vehicle-level metrics confirms that the RL agent not only outperforms the static baseline but also surpasses the standard semi-adaptive strategy used in field deployments. This three-way comparison provides a more rigorous validation than the typical RL-versus-fixed-time evaluations prevalent in the literature.

## 7. Discussion

The experimental results present an interesting duality: while the temporal efficiency (waiting time) showed almost no change, the spatial efficiency (queue length) improved dramatically. This section analyzes these findings, the behavior of the AI agent, and the implications of using simulated IoT data.

### 7.1. Dual-Metric Interpretation: Macro vs. Micro Efficiency

A key finding of this study is the divergence between network-level and vehicle-level metrics. At the macro level, all three controllers achieve an equivalent mean waiting time of 0.91 s, indicating that the UDOT-derived timing plan is already near-optimal for the aggregate demand. At the micro level, however, the AI agent reduces per-vehicle waiting time by 15.5% and total queue length by 12.3%.

This phenomenon can be understood as a difference in *measurement granularity*. The network-level metric averages over all vehicles in the simulation—including those in free-flow far from the intersection—thereby diluting the delay experienced by vehicles directly affected by signal timing. The vehicle-level metric captures the actual delay incurred by each individual trip, making it more sensitive to the quality of signal control decisions.

The actuated controller occupies an intermediate position in this hierarchy, reducing per-vehicle waiting time by 7.3% and queue length by 6.7% relative to the fixed-time baseline. Its gap-based phase extension mechanism provides partial adaptivity: it can extend green time for an approach with continuous vehicle arrivals, but it cannot proactively anticipate demand or redistribute green time across conflicting phases. The AI agent, by contrast, learns a global policy that considers the state of all approaches simultaneously, enabling more effective temporal redistribution.

**Practical Implications:** From an urban planning perspective, the reduction in queue length is particularly significant. Even moderate queue reductions (12.3% in this study) can prevent “spillback”—the blockage of upstream intersections or turn pockets—which is a primary cause of gridlock in dense urban networks. The three-way comparison demonstrates that while actuated control provides incremental improvements over static timing, the RL agent achieves a qualitatively different level of spatial optimization. This aligns with emerging Cyber–Physical Digital Twin architectures that combine hybrid optimization with machine learning for smart city infrastructure [15]. Moreover, recent updates from federal transportation authorities emphasize the transition toward adaptive and data-driven signal control systems as part of long-term infrastructure modernization strategies [16].

### 7.2. Responsiveness to Traffic Variability

The traffic demand profile used (derived from real ATSPM data) is not perfectly smooth; it contains random arrivals and small spikes in volume. As seen in the queue length results (Figure 7), the baseline controller allows queues to build up periodically because it must wait for its pre-programmed cycle to finish. It cannot “see” that a lane is full.

In contrast, the Q-learning agent acts as a reactive system. Emulated IoT sensors provide the agent with a snapshot of the intersection every step. When a sudden platoon arrives, the state value $S_{t}$ changes, and the agent exploits its learned policy to extend the Green time for that specific approach. This adaptability confirms that Reinforcement Learning is particularly useful for handling stochastic (random) traffic patterns that static schedules cannot predict, a finding consistent with recent studies on self-adaptive traffic systems [17].

### 7.3. The Role of the Digital Twin and IoT Emulation

This study relied on a Digital Twin approach where IoT sensors were emulated via SUMO’s TraCI interface. In a real-world deployment, this data would come from inductive loops, radar, or computer vision cameras.

The simulation assumes perfect data availability—the agent knows the exact queue length instantly. In reality, physical IoT sensors have noise, latency, and occlusion (e.g., a large truck hiding a small car). While our results are promising, they represent a “best-case scenario” for sensor accuracy. However, similar Digital Twin validations have shown that AI controllers can maintain robustness even under partial observability [18]. The framework demonstrates that if the data pipeline is fast enough (low latency), the control logic itself is sound. The Digital Twin effectively bridges the gap between theoretical algorithms and physical constraints, allowing us to verify the safety of the AI before it ever touches a real traffic light.

### 7.4. Comparison with State-of-the-Art Methods

To contextualize the performance of the proposed Q-learning agent, Table 5 compiles representative results from recent RL-based traffic signal control studies. Direct comparison of absolute metric values across studies is inherently limited due to differences in network topology, demand patterns, and simulation configurations. Nevertheless, the relative improvements provide a useful reference for evaluating the competitiveness of the proposed approach.

A critical distinction must be emphasised when interpreting these results. The majority of the listed studies compare their RL agents against generic or unoptimized fixed-time controllers, which can inflate the reported improvement margins. In contrast, the present study benchmarks the Q-learning agent against both a professionally engineered timing plan derived from operational UDOT ATSPM data and a vehicle-actuated controller—representing two levels of real-world practice.

Despite this more rigorous evaluation framework, the agent achieves a 15.5% reduction in per-vehicle waiting time and a 12.3% reduction in total queue length relative to the optimized fixed-time plan, and 8.8% and 6.0% respectively relative to the actuated controller. These improvements, while more modest than the large margins reported against unoptimized baselines, are arguably more meaningful as indicators of practical deployment value.

Furthermore, the three-way comparison (Fixed → Actuated → AI) establishes a clear performance hierarchy, demonstrating that the RL agent does not merely replicate the behavior of conventional adaptive controllers but achieves genuine additional optimization through its learned policy.

### 7.5. Limitations and Future Work

While the results are promising, three specific limitations must be acknowledged, which outline the roadmap for future research:

1. **Single Intersection Scope:** The current study focuses on an isolated node. Optimizing a single intersection can sometimes push congestion to neighboring nodes. Future work will extend this framework to a multi-agent corridor to investigate the coordination effects between adjacent Digital Twins.
2. **Idealized Sensing:** The simulation assumes perfect knowledge of queue lengths via TraCI. In physical deployments, sensors suffer from noise and occlusion. Future iterations will introduce stochastic sensor noise and latency into the simulation to test the agent’s robustness against imperfect IoT data.
3. **Pedestrian Interaction:** The current model optimizes strictly for vehicular flow. To ensure viability for real-world urban environments, future experiments will incorporate pedestrian crossing phases and safety constraints into the reward function.

## 8. Conclusions

This paper presented a complete Digital Twin framework for testing AI-based traffic signal control. By integrating real-world data from the UDOT ATSPM system into the SUMO simulator, we created a realistic testing environment for the intersection of State Street and 4500 South.

The experimental results demonstrate that the Q-learning agent successfully learned to manage complex traffic flows. The comparison with both the fixed-time baseline and a vehicle-actuated controller yielded the following key findings:

1. **Superior Vehicle-Level Efficiency:** The AI controller reduced per-vehicle waiting time by 15.5% relative to the fixed-time baseline and by 8.8% relative to the actuated controller, demonstrating that the autonomous agent can surpass not only static plans but also standard semi-adaptive strategies.
2. **Effective Queue Management:** The AI agent reduced total queue length by 12.3% relative to the fixed-time plan and 6.0% relative to the actuated controller, lowering the risk of upstream spillback and demonstrating a critical advantage for space-constrained urban environments.
3. **Preservation of Network-Level Stability:** At the macro level, all three controllers achieved equivalent mean waiting time (0.91 s), confirming that the AI agent does not disrupt overall network efficiency while achieving targeted micro-level improvements.

The three-way comparison (Fixed-Time < Actuated < AI) across all vehicle-level metrics provides a more rigorous validation than the typical RL-versus-fixed-time evaluations prevalent in the literature. Future work will focus on extending this framework to a multi-intersection network. We plan to investigate how multiple AI agents can cooperate to optimize traffic flow along an entire corridor, and how Vehicle-to-Infrastructure (V2I) communication data can further improve decision-making accuracy.

## Figures and Tables

**Table 1:** Comparison of the proposed framework with existing RL-based TSC platforms.

Feature	SUMO-RL	LibSignal	PyTSC	CityFlow	Ours
Simulator(s)	SUMO	SUMO, CF, CB	SUMO, CF	CityFlow	SUMO
RL Scope	S/M	Multi	MARL	Multi	Single
IoT/DT Layer	×	×	×	×	✓
Real-World Data	×	Mixed	Mixed	Mixed	ATSPM
Geometric Calibration	×	×	×	×	✓
Site-Specific Valid.	×	×	×	×	✓

CF = CityFlow; CB = CBEngine; S/M = Single/Multi-agent; ATSPM = Automated Traffic Signal Performance Measures; × = not supported; ✓ = supported.

**Table 2:** Quantitative Validation of Simulation Parameters.

Parameter	Physical Reality (UDOT)	Digital Twin (SUMO)
Lane Configuration	5 lanes (Major)/3 lanes (Minor)	Matched exactly
Speed Limit	35–45 mph	15.6–20.1 m/s
Phase Logic	NEMA 8-Phase Dual Ring	NEMA Compliant
Saturation Flow	≈1900 veh/h/lane	Calibrated (Krauss Model)
Clearance Time (Yellow)	3.5 s	3.5 s

**Table 3:** Network-level performance comparison (macro-metrics from SUMO summary).

Metric	Fixed-Time	Actuated	AI (Q-Learning)
Mean Waiting Time (s)	0.91	0.91	0.91
Mean Halting Vehicles	3.6	3.4	3.1

**Table 4:** Vehicle-level performance comparison (micro-metrics from trip records and queue data).

Metric	Fixed-Time	Actuated	AI (Q-Learning)	AI vs. Fixed (%)
Avg. Waiting Time (s)	9.18	8.51	7.76	**−15.5%**
Avg. Queue Length (veh)	21.83	20.36	19.14	**−12.3%**
Throughput (trips)	2535	2537	2538	≈0.0%

Bold values indicate the primary improvement metrics of the AI controller relative to the fixed-time baseline.

**Table 5:** Representative RL-based TSC results from recent literature.

Study	Method	Scope	Baseline	Metric	Improv.
Guo et al. (2019) [19]	DQN	Single	FT, SOTL, Act.	Queue	27–73%
Pande (2023) [20]	DQN	Single	Fixed-Time	Wait/Queue	8.5/18.5%
Hu et al. (2024) [9]	MA-DDQN	Corridor	FT, PASSER-V	Wait/Queue	40–60%
Bouali et al. (2025) [21]	SA + RL	Single	Fixed-Time	Wait	21.6%
Zhong et al. (2023) [22]	DQN (V2I)	Single	Fixed-Time	Wait	≈40%
Mortazavi Azad et al. (2023) [23]	Q-Learning	Single	Fixed-Time	Queue time	34%
**This work**	**Q-Learn.**	**Single**	**Optim. + Act.**	**Wait/Queue**	**15.5/12.3%**

FT = Fixed-Time; Act. = Actuated; SA = Simulated Annealing; Optim. = Optimized UDOT plan. Bold highlights the present study for visual distinction from the literature entries. All studies use SUMO.

## Data Availability

The data used in this study are available from the corresponding author upon reasonable request.

## Abbreviations

The following abbreviations are used in this manuscript:

AI	Artificial Intelligence
IoT	Internet of Things
ITS	Intelligent Transportation Systems
RL	Reinforcement Learning
SUMO	Simulation of Urban MObility
TraCI	Traffic Control Interface
UDOT	Utah Department of Transportation
ATSPM	Automated Traffic Signal Performance Measures
TMC	Turning Movement Counts
NEMA	National Electrical Manufacturers Association
V2I	Vehicle-to-Infrastructure
ML	Machine Learning
